# Base rates, blindness, and schizophrenia

**DOI:** 10.3389/fpsyg.2013.00157

**Published:** 2013-04-03

**Authors:** Steven M. Silverstein, Yushi Wang, Matthew W. Roché

**Affiliations:** ^1^ University Behavioral HealthCare, University of Medicine and Dentistry of New Jersey Piscataway, NJ, USA; ^2^ Department of Psychiatry, Robert Wood Johnson Medical School, University of Medicine and Dentistry of New Jersey Piscataway, NJ, USA

Our paper on congenital and early (C/E) blindness and schizophrenia was an effort to account for the previously reported negative relationship between these conditions, in light of recent work indicating what the study of C/E blind people reveals about brain function and organization (Cattaneo and Vecchi, 2011; Kupers et al., 2011; Ricciardi and Pietrini, 2011). In particular, we hypothesized that the neuroplastic changes inherent to C/E blindness protect against schizophrenia by promoting strengths in those cognitive domains where schizophrenia is characterized by deficits.

Since our paper was published, we have received several comments asking a version of this question: can the lack of reports of people with both conditions be explained simply by the rarity of each condition, so that the chances of a person having both are so low that even if such people existed, it is unlikely their condition will have been reported? This is an important question, as when studying low base rate phenomena, distorted conclusions can easily be reached (Meehl and Rosen, 1955). We addressed this issue in the original version of our manuscript, but then removed it from the final version due to length considerations. Here, we note our earlier comments.

The conclusion that there are no C/E blind people with schizophrenia is based on a small number of studies that involved relatively small samples. Clearly, this argument would be strengthened by larger, population-based studies. This is because, as a simple calculation demonstrates, a case of congenital blindness and schizophrenia would be extremely rare even if there was no protective effect of blindness: if schizophrenia occurs at a rate of 0.72% in the population (McGrath et al., 2008) and congenital blindness occurs at an estimated rate of 0.03% in people born in the 1970s and 1980s (based on Robinson et al., 1987), then the joint probability of a person having both conditions, if the two are independent, would be 0.0002% or 2 out of every 1 million people. Although this is a low prevalence rate, it is equal to or higher than the rates for several other well-known conditions (e.g., Creutzfeldt-Jakob disease, hereditary spastic paraplegia, Hermansky-Pudlak Syndrome). Based on this estimated prevalence rate, in the United States alone (with a population of 311, 591, 917, as of July 2011, according the US census), there should be approximately 620 congenitally blind people with schizophrenia. When cases of blindness with an onset in the first year of life (i.e., early blindness) are taken into account, the percentage would be larger. Therefore, it is remarkable that in over 60 years, and with several investigations [including several before DSM-III (1980) when criteria for schizophrenia were broader than at present], not a single case of a C/E blind schizophrenia patient has been reported. Moreover, several published studies, and our experience as well, included surveying Directors of agencies that serve large numbers of blind people, and none of them could recall ever seeing a person who had both conditions. It is also interesting that rates of C/E blindness are significantly higher in developing, compared to industrialized, countries. Therefore, if C/E blindness did not protect against the development of schizophrenia, comorbidity would be more likely to be reported in such countries. However, this has not occurred. In short, available evidence, probabilistic estimates, and the striking contrasts, within the same domains of cognition, between superior functioning in C/E blindness and impaired functioning in schizophrenia, combine to suggest a protective relationship. If the conditions did co-occur at chance levels, reports of such cases should appear at least somewhat as often as those of many other rare medical conditions, especially since reports of an absence of schizophrenia in C/E blind people have appeared since 1950 (Chevigny and Braverman, 1950). The absence of such reports is noteworthy.

One research strategy that could generate further evidence on this issue is the study of schizotypal symptoms in the C/E blind. These subsyndromal psychotic symptoms have a higher base rate than schizophrenia itself (i.e., 10–15% vs. ~1%) (Fossati and Lenzenweger, 2009), and people with these symptoms share some of the same cognitive and biological impairments as people with the full disorder (Aichert et al., 2012; Cochrane et al., 2012). Studies of schizotypal symptoms would allow for the determination of whether C/E blindness protects against the full spectrum of schizophrenia-related illness or just schizophrenia itself. If no schizotypal symptoms were observed, this would be evidence for the existence of protective mechanisms against all schizophrenia-related psychopathology. If, however, schizotypal symptoms were found, samples of C/E blind people could be studied to gain insight into the neurobiological changes that protect against the development of clinical psychosis. As such, expanding the schizophrenia phenotype studied in the C/E blind holds potential for increasing our understanding of the mechanisms involved in schizophrenia.

In addition to these points, we noted in our original paper that other congenital sensory impairments have not been associated with absence of schizophrenia, that congenital deafness and later blindness, such as found in Usher Syndrome, whose prevalence has been estimated at 0.005% (Rosenberg et al., 1997) has been associated with psychotic disorders at rates between 4–24% (Waldeck et al., 2001), and that non-psychotic psychiatric disorders are observed in C/E blindness. These data suggest a unique relationship between C/E blindness and schizophrenia. However, we acknowledge that the absence of evidence (of people with both conditions) is not evidence of absence. That said, given the potentially important lessons for understanding, preventing, and treating schizophrenia that brain reorganization in C/E blindness provides, we believe it is useful to pursue this line of thought. At the very least, if C/E blindness did *not* prevent schizophrenia, then in light of the cognitive changes secondary to C/E blindness, it would be informative to determine whether it mitigates the associated cognitive impairment. Finally, we hope to raise awareness in the psychiatric community of past literature relating to C/E blindness and schizophrenia, so that if people with both conditions exist, their cases will be reported.
