# Corrigendum: Base Rates, Blindness, and Schizophrenia

**DOI:** 10.3389/fpsyg.2021.732333

**Published:** 2021-09-21

**Authors:** Steven M. Silverstein, Yushi Wang, Matthew W. Roché

**Affiliations:** ^1^University Behavioral HealthCare, University of Medicine and Dentistry of New Jersey, Piscataway, NJ, United States; ^2^Department of Psychiatry, Robert Wood Johnson Medical School, University of Medicine and Dentistry of New Jersey, Piscataway, NJ, United States

**Keywords:** schizophrenia, blindness, cognition, perception, vision, plasticity, vision disorders

In the original article there was an error. The text stated that: “…if schizophrenia occurs at a rate of 0.72% in the population (McGrath et al., 2008), and congenital blindness occurs at an estimated rate of 0.03% in people born in the 1970s and 1980s (based on Robinson et al., 1987), then the joint probability of a person having both conditions, if the two are independent, would be 0.02% or 2 out of every 10,000. Although this is a low prevalence rate, it is higher than the rates for childhood-onset schizophrenia (Remschmidt and Theisen, 2005), and many other well-known medical conditions (e.g., Hodgkin's lymphoma, Prader Willi syndrome, Rett's Syndome).”

This joint probability estimate was reported incorrectly. The joint probability is not the product of the percentages; it should be the product of the corresponding decimal values (.0072 ^*^ .0003). Therefore, the correct value should be 0.0000021 (or .00021%). In addition, the corrected joint probability rate is now lower than the prevalence rate for the diseases cited. Nevertheless, the joint probability of schizophrenia and congenital/early blindness is still equal to or higher than a number of well-known rare diseases, and examples are cited in the corrected text. Note that although the joint probability rate was changed, the expected prevalence rate discussed in the following sentences was stated correctly in the original publication.

A correction has been made to *Paragraph 3*. The corrected text is shown below.

The conclusion that there are no C/E blind people with schizophrenia is based on a small number of studies that involved relatively small samples. Clearly, this argument would be strengthened by larger, population-based studies. This is because, as a simple calculation demonstrates, a case of congenital blindness and schizophrenia would be extremely rare even if there was no protective effect of blindness: if schizophrenia occurs at a rate of 0.72% in the population (McGrath et al., 2008), and congenital blindness occurs at an estimated rate of 0.03% in people born in the 1970s and 1980s (based on Robinson et al., 1987), then the joint probability of a person having both conditions, if the two are independent, would be 0.0002% or 2 out of every 1 million people. Although this is a low prevalence rate, it is equal to or higher than the rates for several other well-known conditions (e.g., Creutzfeldt-Jakob disease, hereditary spastic paraplegia, Hermansky-Pudlak Syndrome). Based on this estimated prevalence rate, in the United States alone (with a population of 311, 591, 917, as of July 2011, according the US census), there should be approximately 620 congenitally blind people with schizophrenia. When cases of blindness with an onset in the first year of life (i.e., early blindness) are taken into account, the percentage would be larger. Therefore, it is remarkable that in over 60 years, and with several investigations [including several before DSM-III (1980) when criteria for schizophrenia were broader than at present], not a single case of a C/E blind schizophrenia patient has been reported. Moreover, several published studies, and our experience as well, included surveying Directors of agencies that serve large numbers of blind people, and none of them could recall ever seeing a person who had both conditions. It is also interesting that rates of C/E blindness are significantly higher in developing, compared to industrialized, countries. Therefore, if C/E blindness did not protect against the development of schizophrenia, comorbidity would be more likely to be reported in such countries. However, this has not occurred. In short, available evidence, probabilistic estimates, and the striking contrasts, within the same domains of cognition, between superior functioning in C/E blindness and impaired functioning in schizophrenia, combine to suggest a protective relationship. If the conditions did co-occur at chance levels, reports of such cases should appear at least somewhat as often as those of many other rare medical conditions, especially since reports of an absence of schizophrenia in C/E blind people have appeared since 1950 (Chevigny and Braverman, 1950). The absence of such reports is noteworthy.

Additionally, the reference “Remschmidt and Theisen, 2005” has been removed from the article, as it is no longer needed.

The correspondence email address has also been updated.

The authors apologize for these errors and state that they do not change the scientific conclusions of the article in any way. The original article has been updated.

## Publisher's Note

All claims expressed in this article are solely those of the authors and do not necessarily represent those of their affiliated organizations, or those of the publisher, the editors and the reviewers. Any product that may be evaluated in this article, or claim that may be made by its manufacturer, is not guaranteed or endorsed by the publisher.
